# Supplementary material for: Polymorphisms in Pfkelch13 domains before and after the introduction of artemisinin-based combination therapy in Southwest Nigeria
Source: PLoS One. 2025 Mar 31;20(3):e0316479. doi: 10.1371/journal.pone.0316479 (PMC11957316; doi:10.1371/journal.pone.0316479)
Supplement: Supporting information 2 — (ZIP) [file pone.0316479.s002.zip › 133KNIFW_PREMIX_Plate_CORKELCH_H09.pdf]

Samples: 12370
Bases: 603
Average spacing: 21

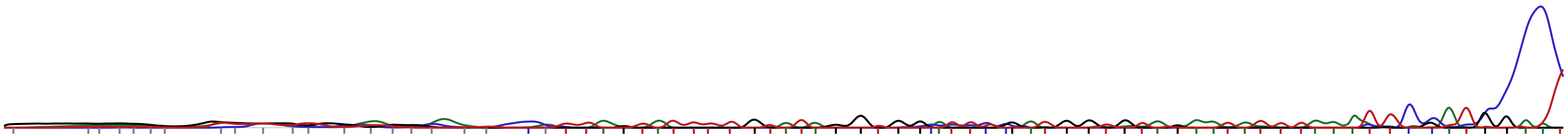

- - - - - - - - - - - - - - - - - - - C - T T A G T A T T T T G T A T A G G T G G C A T T C C G A T G G T G T A G A A T A T T T A A A T T C C A T G G A A T

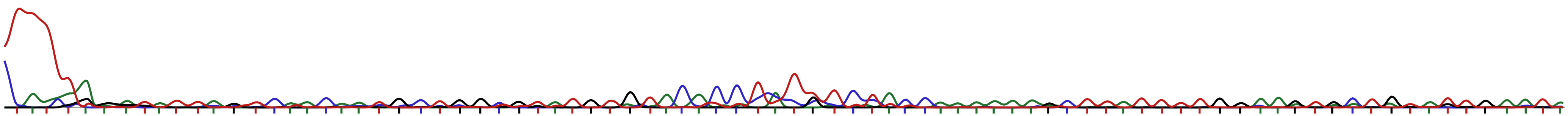

T A T T A A A T A T T A G T C A A C A A T G C T G G C G T A T G T G T A C A C C T A T G T C T A C C A A A A A A G C T T A T T T T G G A A G T G C T G T A T T G A A T A

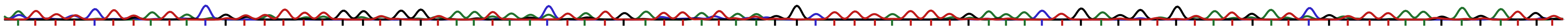

A T T T C T T A T A C G T T T T T G G T G G T A A T A A C T A T G A T T A T A A G G C T T T A T T T G A A A C T G A G G T G T A T G A T C G T T T A A G A G A T G T

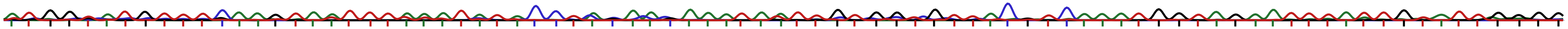

A T G G T A T G T T T C A A G T A A T T T A A A T A T A C C T A C A A C A A A T A A T T G T G G T G T T A C G T C A A A T G G T A G A A T T T A T T G T A T T G G G G

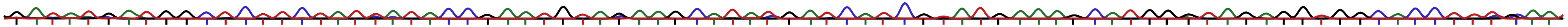

G A T A T G A T G G C T C T T C T A T T A T A C C G A A T G T A G A A G C A T A T G A T C A T C G T A T G A A A G C A T G G G T A G A G G T G G C A C C T T T C A A

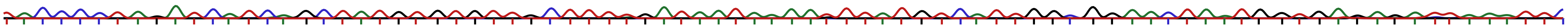

T A C C C C T A G A T C A T C A G C T A T G T G T G T T G C T T T T G A T A A T A A A A T T T A T G T C A T T G G C G G A A C T A A T G G T G A G A G A T T A A A T T C

Samples: 12370
Bases: 603
Average spacing: 21

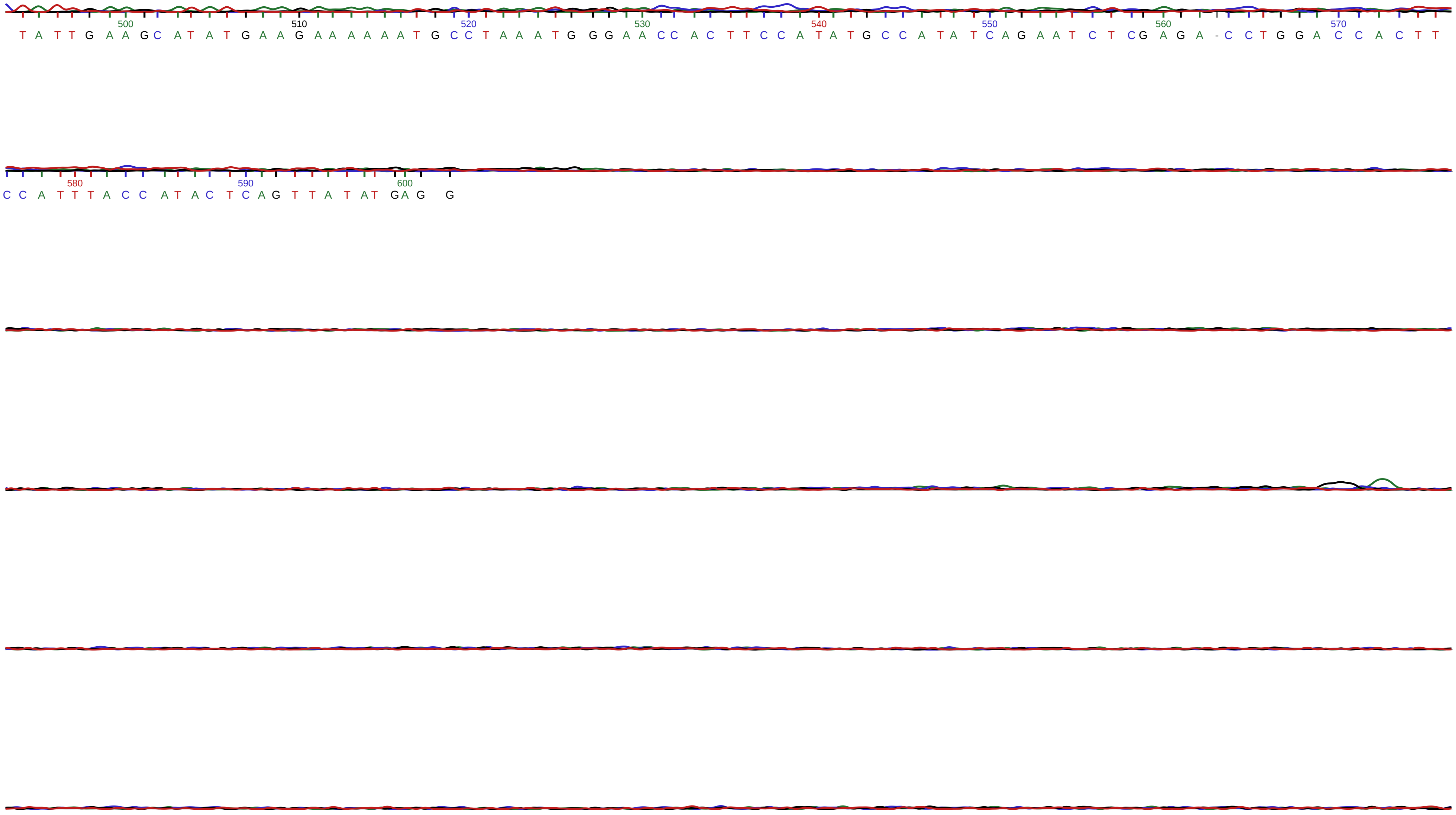

Samples: 12370
Bases: 603
Average spacing: 21
